# Pleural Mesothelioma With Unusual Squamous Differentiation: A Clinicopathological Case Report With Molecular Insights From Whole‐Exome Sequencing

**DOI:** 10.1111/pin.70190

**Published:** 2026-10-07

**Authors:** Julius Bodemeyer, Mara Elisa Mierzwa, Jörg Seraphin, Rizkallah Maroun, Wolfgang Körber, Christoph Engelke, Felix Bremmer, Tessa Rosenthal, Tim Heißenberg, Kirsten Reuter‐Jessen, Philipp Ströbel, Alexander Marx, Hanibal Bohnenberger

**Affiliations:** ^1^ Institute of Pathology University Medical Center Göttingen Germany; ^2^ Medical Care Center Onkologikum Northeim Northeim Germany; ^3^ Department for Pneumology Evangelical Hospital Göttingen‐Weende Göttingen Germany; ^4^ Department for Radiology Evangelical Hospital Göttingen‐Weende Göttingen Germany; ^5^ Department of Pathology and Molecular Pathology University Hospital Zurich Zurich Switzerland

**Keywords:** differential diagnosis, mesothelioma, squamous transdifferentiation, whole‐exome sequencing

## Abstract

Pleural mesothelioma is an aggressive tumor with a poor prognosis and limited therapeutic options. While histological heterogeneity is common, true squamous differentiation is extremely rare and diagnostically challenging. We report an 81‐year‐old man with asbestos exposure who presented with recurrent right‐sided pleural effusion. Thoracoscopic biopsies revealed two distinct tumor components. The first expressed mesothelial immunohistochemical markers, whereas the second showed a squamous profile. Both components demonstrated loss of BAP1 expression and homozygous deletion of CDKN2A, supporting a common mesothelial origin. Whole‐exome sequencing revealed a tumor mutational burden above the average reported for mesothelioma (8–9 mutations per megabase), with 69 somatic mutations in total, 43 shared between the two components. In addition to canonical mesothelioma alterations (BAP1, CHEK2), the squamous component harbored 18 unique variants, including oncogenic alterations in NFE2L2 and PIK3CA. A novel SOX9 missense variant of uncertain significance (p. Thr243Pro) was also identified within the transactivation middle (TAM) domain; its functional consequence remains unknown. This case highlights a rare histological variant of pleural mesothelioma with partially squamous differentiation, characterized by a mutational burden above the mesothelioma average and squamous carcinoma‐associated alterations. Integrated histology, immunohistochemistry, and genomic profiling are essential to avoid misdiagnosis and to improve understanding of tumor plasticity in mesothelioma.

## Graphical abstract

This case report highlights a rare histological variant of pleural mesothelioma with partially squamous differentiation in an 81‐year‐old man with occupational asbestos exposure. In addition to histological, immunohistochemical and fluorescence in situ hybridization analyses, indicating a common mesothelial origin of both tumor components, whole‐exome sequencing was performed separately on both components. This enabled a comparison of their molecular profiles and exploration of potential mechanisms underlying squamous transdifferentiation.

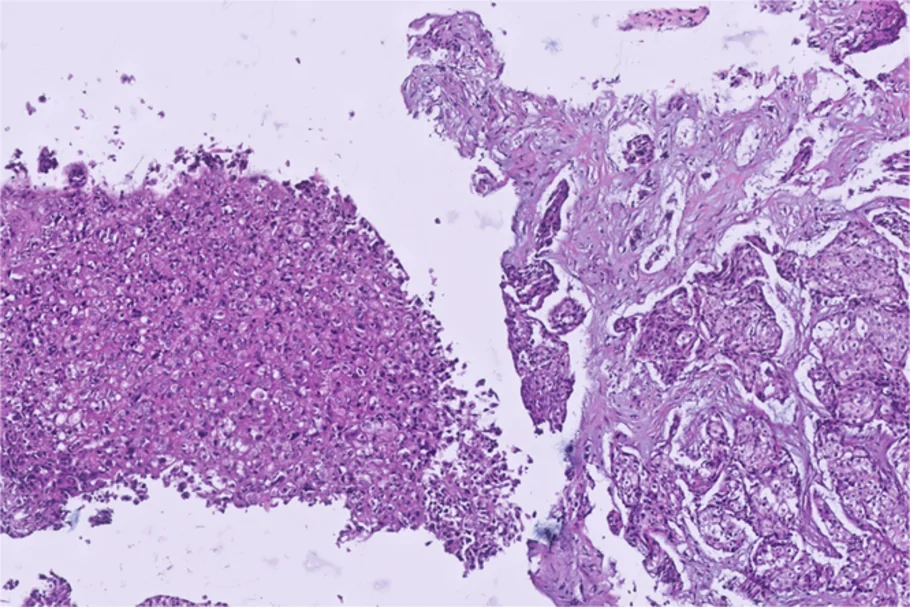

**Abbreviations**
BAP1: BRCA1‐associated protein 1
CDKN2A: cyclin dependent kinase inhibitor 2 A
CEA: carcinoembryonic antigen
CHEK2: checkpoint kinase 2
CK: cytokeratin
CT: computed tomography
EZH2: enhancer of Zeste Homolog 2
FISH: fluorescence in situ hybridization
MTAP: methylthioadenosine phosphorylase
PD‐L1: programmed death‐ligand 1
SCC: squamous cell carcinoma
SOX9: SRY‐Box Transcription Factor 9
TMB: tumor mutational burden
TPS: tumor proportion score
TTF‐1: thyroid transcription factor 1
WES: whole‐exome sequencing
WT1: Wilms tumor protein

## 1 Introduction

Mesothelioma is a rare and highly aggressive neoplasm with an estimated incidence of about 0.2% among all malignancies [1]. More than 80% of cases arise in the pleura. The principal cause of mesothelioma is asbestos exposure [2, 3]. Mesothelioma is histologically divided into epithelioid, biphasic, and sarcomatoid subtypes, with proportions of roughly 40%–60%, 20%–30%, and 20%–30% respectively [3]. Median overall survival ranges from 8 to 36 months depending on stage and histological subtype [4].

Because of overlapping morphology with reactive mesothelial proliferations or other malignancies, immunohistochemistry is essential for establishing mesothelial origin and assessment of malignancy. Panels typically include mesothelial markers such as calretinin, WT1 (Wilms tumor protein), D2‐40, and CK 5/6 (Cytokeratin 5/6), and epithelial markers such as claudin‐4, BerEP4/MOC31, CEA (Carcinoembryonic antigen), and B72.3 [3, 5]. However, the expression of mesothelial markers may be weak or lost completely, especially in the sarcomatoid subtype [3]. Additional diagnostic tools help to distinguish malignant from reactive processes. Immunohistochemical loss of BAP1 (BRCA1‐associated protein 1) or MTAP (methylthioadenosine phosphorylase) expression, homozygous CDKN2A (cyclin dependent kinase inhibitor 2 A) deletion by fluorescence in situ hybridization (FISH), and EZH2 (Enhancer of Zeste Homolog 2) overexpression all provide important diagnostic support [3, 6].

Pleural mesotheliomas present with a wide range of histological patterns, for example, solid or tubulopapillary growth or spindle cell morphology [3]. The recognition of this morphological diversity is essential, as it may complicate diagnosis and sometimes mimic other tumor entities. Within this spectrum, squamous differentiation is exceedingly rare. Only a few reports have documented mesotheliomas with a squamous component [7, 8, 9, 10].

Against this background, we report a case of epithelioid pleural mesothelioma with a distinct squamous component, diagnosed from a relatively small pleural biopsy. In contrast to prior reports, we additionally performed whole‐exome sequencing (WES) on both tumor components, which enabled us to compare their molecular profiles and to explore potential mechanisms underlying squamous transdifferentiation. To our knowledge, this is the first report combining detailed histopathological, immunohistochemical, and genomic analyses of such a constellation.

## 2 Clinical Summary

An 81‐year‐old man presented with a right‐sided pleural effusion of 2 L. Computed tomography (CT) showed pleural thickening and multiple micronodules in the posterior upper lobe of the right lung (Figure 1). His past medical history included pulmonary tuberculosis in childhood. He had never smoked but reported occupational asbestos exposure for 8 years. The pleural effusion recurred within 4 weeks, prompting further diagnostic evaluation. Thoracoscopy revealed multiple plaques and polypoid changes along the chest wall and diaphragm.

**Figure 1:**
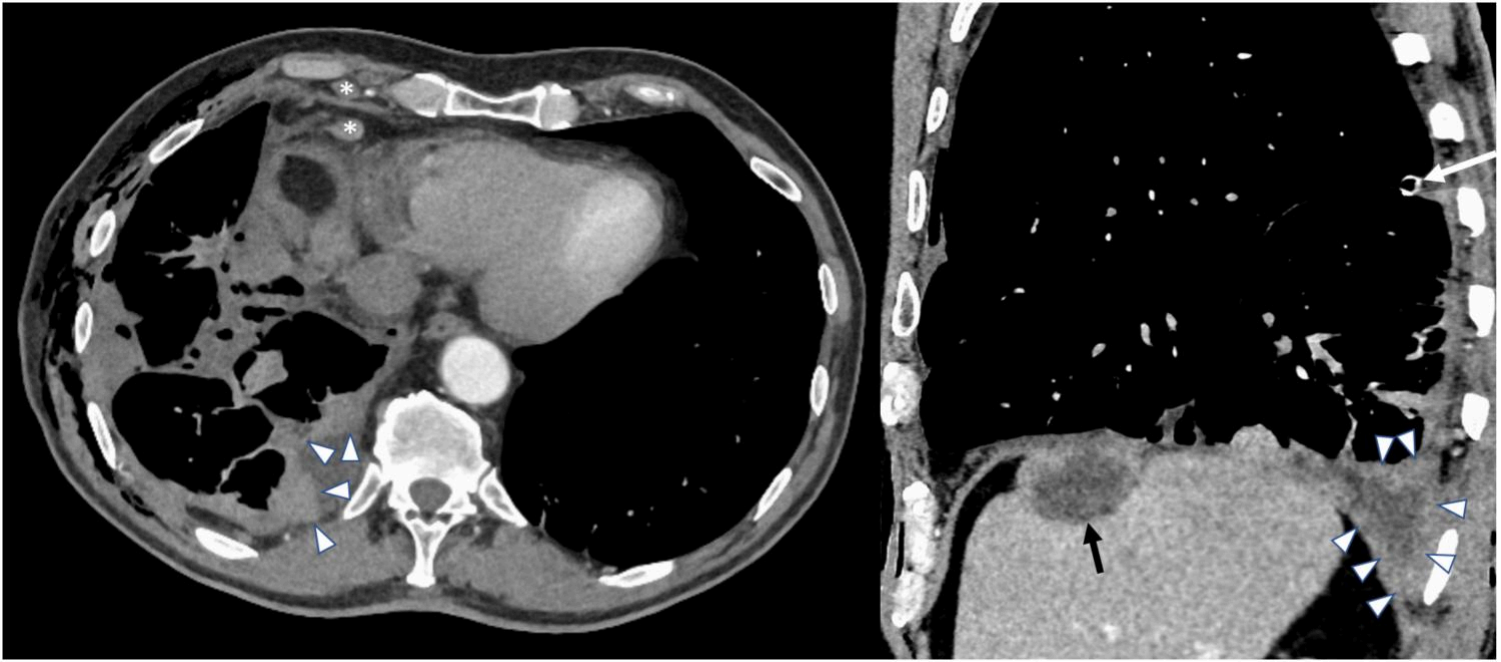
Computed tomography scan at initial presentation. Contrast‐enhanced computed tomography of the chest after drainage of the right pleural effusion showing the drainage tube (white arrow), a loculated residual effusion with multiple basal areas of gross pleural thickening (white arrowheads) and suspicious pararecessal epipleural and extrapleural lymphadenopathy (asterisks). There is superior compression of the liver capsule by a partially liquid pleural mass (black arrow).

## 3 Pathological Findings

During thoracoscopy, biopsies were obtained, which showed fibrotic pleura with two distinct populations of atypical cells. The first consisted of epithelioid cells with pleomorphic, hyperchromatic nuclei and amphophilic, partly clear cytoplasm. The second population displayed a solid growth pattern with more distinct cell borders, paler cytoplasm and smaller nuclei.

Immunohistochemistry further underscored the biphasic appearance (Table 1; Figures 2 and 3). The first component showed positivity for calretinin, GATA3, and CK7, with weak focal staining for CK5/6, D2‐40 and WT1, while p40 and p63 were negative. In contrast, the second component was strongly positive for p40, p63, and CK5/6, and negative for calretinin, GATA3, CK7, D2‐40 and WT1. Proliferative activity, measured by Ki‐67 staining, was high in both tumor components—80% and 70%, respectively. Both components were positive for pancytokeratin and CK8/18, but negative for BerEP4, CEA (Carcinoembryonic antigen) and TTF‐1 (Thyroid transcription factor 1). Importantly, both showed loss of BAP1 expression. FISH analysis targeting the CDKN2A locus demonstrated homozygous deletion in both tumor components (Figure 3).

**Table 1:** Immunohistochemical comparison of both tumor components.

Marker	Mesothelial‐like component	Squamous‐like component
Calretinin	Positive	Negative
GATA3	Positive	Negative
CK7	Positive	Negative
D2‐40	Focal, weak	Negative
WT‐1	Focal, weak	Negative
CK5/6	Focal, weak	Strongly positive
p40	Negative	Strongly positive
p63	Negative	Strongly positive
CD15	Negative	Positive
TROP2	Negative	Positive
PD‐L1 (TPS)	~80%	~5%
Ki‐67	~80%	~60%–70%
Pancytokeratin	Positive	Positive
CK8/18	Positive	Positive
BerEP4	Negative	Negative
CEA	Negative	Negative
TTF‐1	Negative	Negative
BAP1	Loss	Loss
EZH2	Positive (increased expression)	Positive (increased expression)

**Figure 2:**
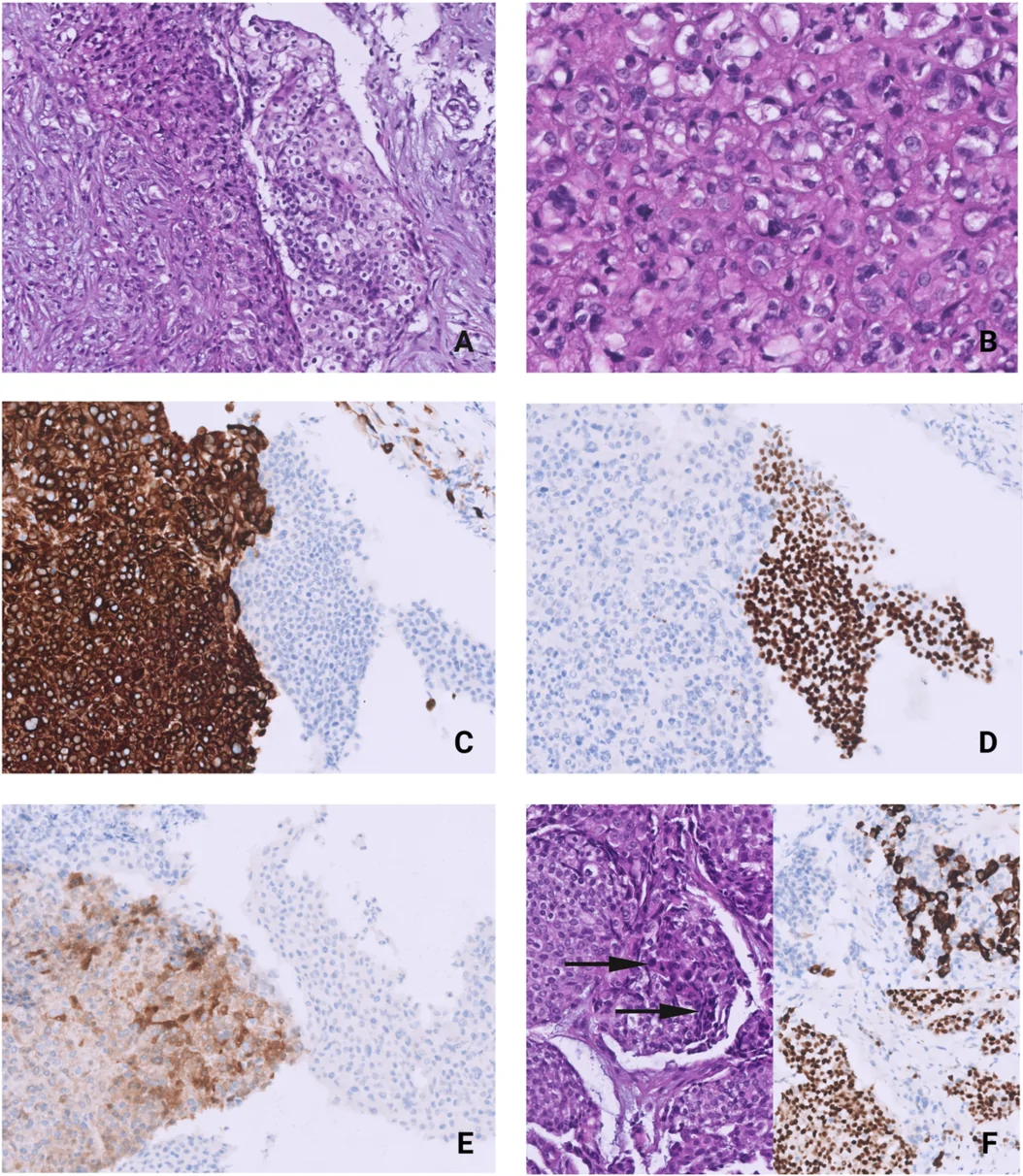
Histological and immunohistochemical comparison of both tumor components. Panel A shows atypical mesothelial cells with an epithelioid morphology on the left and atypical squamous cells with a solid growth pattern on the right. In the middle the two areas are closely adjacent to each other (HE, 200× magnification). Panel B shows the mesothelial component in a higher magnification. The tumor cells show an epithelioid morphology with pleomorphic hyperchromatic nuclei and an amphophilic, partly clear cytoplasm (HE, 400× magnification). In panels C to E, the mesothelial cells (left on each panel) are strongly positive for CK7 (Panel C, 200× magnification), negative for p40 (Panel D, 200× magnification) and positive for Calretinin (Panel E, 200× magnification), whereas the squamous component (right on each panel) presents an opposite immunophenotype. Panel F shows a small area where the two tumor components are admixed with each other. There are some larger mesothelial cells (black arrows) that lie in between sheets of squamous cells (left side of the panel, HE, 200× magnification). Again, these cells are positive for CK7 (upper right, 200× magnification) and negative for p40 (lower right, 200× magnification).

**Figure 3:**
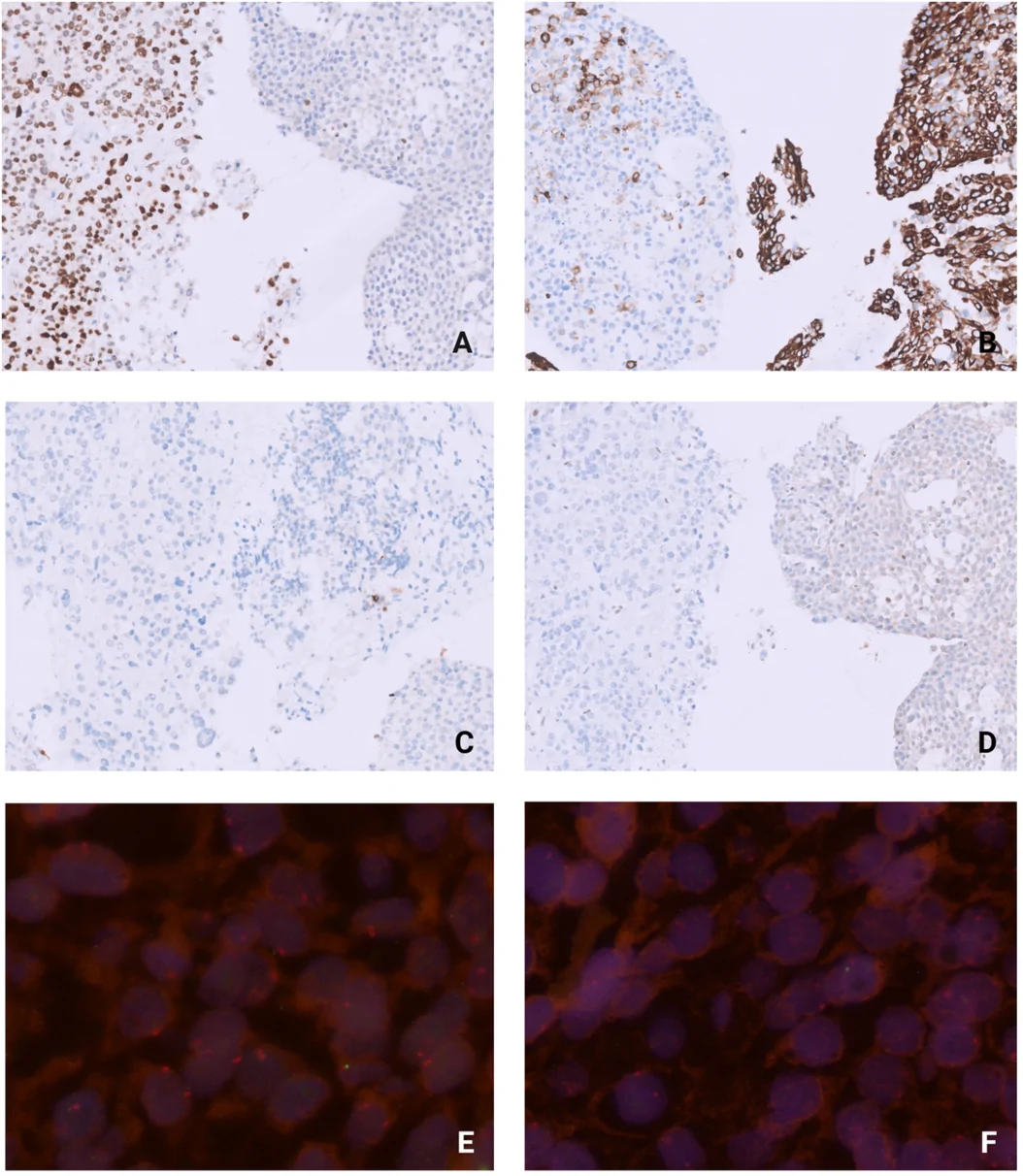
Additional immunohistochemical stains and fluorescence in situ hybridization of both tumor components. In Panel A, the mesothelial cells (left) are positive for GATA3, whereas the squamous cells (right) are negative (200× magnification). Panel B shows immunohistochemical staining for CK5/6. The squamous cells (right) are strongly positive, whereas the mesothelial cells (left) present a focal and weak staining (CK5/6, 200× magnification). Both components are negative for CEA (Panel C, 200× magnification) and show a loss of BAP1 (Panel D, 200× magnification). For BAP1, there a single positive cells (lymphocytes) between the tumor cells, serving as an internal control. Fluorescence in situ hybridization shows homozygous deletion of CDKN2A in the mesothelial component (Panel E, 630× magnification) and squamous component (Panel F, 630× magnification).

On higher magnification, the component with expression of mesothelial markers showed marked nuclear atypia consistent with high‐grade epithelioid mesothelioma; its cohesive epithelioid cytomorphology, without a spindle‐cell or storiform pattern, together with diffuse pancytokeratin and CK8/18 expression, argued against sarcomatoid differentiation. However, the overall tumor architecture could not be assessed because of the limited biopsy material.

Whole‐exome sequencing (WES) was conducted separately for both components. The two components were marked on H&E‐ and p40‐stained reference sections and separately macrodissected from serial unstained sections before DNA extraction, ensuring component‐specific sequencing. In total, 69 somatic mutations were identified, of which 43 were shared by both. The mesothelial component harbored 51 mutations in total, including eight exclusive variants, while the squamous component contained 61 mutations, 18 of which were unique. Among all detected mutations, 14 were classified as likely pathogenic or oncogenic: eight were shared between both components, two were exclusive to the mesothelial component, and four were found only in the squamous component. Tumor mutational burden (TMB) was 8.1 mutations per megabase in the mesothelial component and 9.5 mutations per megabase in the squamous component. Of particular interest were the mutations exclusive to the squamous component. Four were judged as likely pathogenic or oncogenic: NFE2L2, PIK3CA, ANK3, and FIG. 4 (see Table 2 and Section 4).

**Table 2:** Overview of the 14 detected likely pathogenic or oncogenic mutations in both components.

Gene (variant)	Mesothelial‐like component	Squamous‐like component
BAP1 (c.1891‐6_1900del)	x	x
BAP1 (c.228del)	x	x
CHEK2 (c.470T > C)	x	x
MKS1 (c.1408‐34_1408‐6del)	x	x
PROKR2 (c.254G > A)	x	x
TBL1XR1 (c.422_423del)	x	x
VWA1 (c.62_71dup)	x	x
PTPRD (c.5534+7_5534+8ins)	x	x
HLA‐A (c.539_540insCA)	x	
LATS1 (c.2585_2593+22del)	x	
ANK3 (c.5582C > T)		x
FIG. 4 (c.122T > C)		x
NFE2L2 (c.77A > C)		x
PIK3CA (c.1633G > A)		x
SOX9 (p.Thr243Pro)^a^		x

^a^ SOX9 (p.Thr243Pro) is a novel variant of uncertain significance, detected only in the squamous component.

Based on the histological, immunohistochemical and molecular findings and clinical presentation, medical history and imaging findings, we diagnosed high grade epithelioid pleural mesothelioma, with an unusual partially squamous differentiation.

Magnetic resonance imaging of the brain revealed no intracranial metastases. Computed tomography of the abdomen also showed no metastases, although there was suspicion of tumor extension into the right hepatic lobe. Based on these findings, first‐line immunotherapy with nivolumab and ipilimumab was initiated.

Three months later, follow‐up CT imaging demonstrated progressive disease with increased pleural thickening and marked calcifications, and direct invasion through the diaphragm into the right hepatic lobe. Enlarged mediastinal lymph nodes were also noted, although no abdominal metastases were detected. Considering disease progression, a switch to platinum‐based chemotherapy with pemetrexed and carboplatin was initiated. At 7 months after the initial diagnosis, the patient remained alive.

## 4 Discussion

Mesothelioma is characterized by considerable morphological heterogeneity, yet squamous differentiation remains an exceptional finding. To date, only a handful of comparable cases have been reported, most of them based on larger tissue samples obtained at autopsy or surgical resection. The present case is, to our knowledge, the first to establish this diagnosis from a small pleural biopsy while at the same time providing comprehensive genomic profiling of both tumor components. This constellation allows novel insights into the biology of squamous transdifferentiation in mesothelioma.

The mentioned earlier reports serve as useful points of comparison. Matsuo and colleagues in 1993 [7] described a peritoneal mesothelioma with squamous features and discussed that chronic inflammation and the intrinsic plasticity of mesothelial cells, which are capable of cytoskeletal remodeling and lineage shifts, may drive mesothelial cells towards squamous metaplasia. All more recently published cases [8, 9, 10] displayed biphasic morphology with a distinct squamous‐like component. As in our case, the squamous areas were negative for mesothelial markers but expressed classical squamous markers such as p40 and p63. Importantly, immunohistochemical or molecular findings supported a common mesothelial origin of both components, including loss of BAP1 expression or deletion of CDKN2A. The parallels with our findings reinforce the interpretation that squamous differentiation represents a true, albeit rare, phenotypic variant of mesothelioma.

Notably, in our case the mesothelial component showed only focal and weak expression for some classical mesothelial markers (D2‐40, WT1 and CK5/6) which caused severe difficulties in the initial diagnostic workup. However, in total, the other diagnostic findings (positivity for calretinin and GATA3, loss of BAP1 expression, homozygous CDKN2A deletion and the detected mutations including the canonical mesothelioma drivers BAP1 and CHEK2) were sufficienct to assess the mesothelial component as “mesothelial.”

Differential diagnosis is particularly challenging in limited biopsy material, as the squamous component alone could easily be misinterpreted as squamous cell carcinoma (SCC). In our case, several lines of evidence strongly argued against this possibility. First, clinical presentation with reported asbestos exposure and imaging findings were suspicious for mesothelioma and there was no history of SCC elsewhere in the body. Moreover, both components showed loss of BAP1 expression and homozygous CDKN2A deletion. Given that BAP1 loss is exceedingly rare in non‐small cell lung cancer [11], the present BAP1 loss in both components can be interpreted as highly specific for the common mesothelial origin of both components in this context. In addition, whole‐exome sequencing revealed many shared mutations in both components, along with some canonical mesothelioma driver mutations such as BAP1 and CHEK2 [12, 13]. These findings substantiate the conclusion that the tumor originated from a common mesothelial clone with subsequent divergent differentiation. A collision of two biologically independent neoplasms—for example a mesothelioma combined with a squamous cell carcinoma of pulmonary or thymic origin—is rendered very unlikely by these shared clonal alterations, which two independent tumors would not be expected to share. Wenonetheless, that the diagnosis was based on small thoracoscopic biopsies and the overall tumor architecture could therefore not be assessed.

While mesotheliomas generally display a low somatic mutational burden, averaging < 2 mutations per megabase, our case demonstrated 8.1 mutations per megabase in the mesothelial component and 9.5 mutations per megabase in the squamous component, respectively. Such above‐average levels of mutational burden are reported in < 5% of cases in large genomic series [12, 13, 14]. Although clearly above the average for mesothelioma, these values remain below the conventional pan‐tumor threshold for high tumor mutational burden (≥ 10 mutations per megabase) and should therefore not be regarded as TMB‐high. It is conceivable that this above‐average mutational load increased tumor plasticity and created a permissive genomic environment for divergent lineage differentiation. Consistent with this, the tumor progressed early under combined immune‐checkpoint inhibition: neither tumor mutational burden nor PD‐L1 expression are validated predictors of benefit from immunotherapy in pleural mesothelioma, and the marked intratumoural heterogeneity of PD‐L1 expression between the two components (approximately 80% vs. 5%) may have further contributed to immune escape.

In total, 69 somatic mutations were identified, of which 43 were shared between both components, reflecting their common origin. Particularly noteworthy were the 18 mutations unique to the squamous component, four of which were classified as likely pathogenic or oncogenic: NFE2L2, PIK3CA, ANK3 and FIG. 4 (Table 2 and Additional files S1 and S2 for all detected variants). The genomic findings should be interpreted with some caution, as the slightly limited quality of the DNA sequencing (for the mesothelial component) obtained from the small biopsy may affect variant calling.

Activating mutations of nuclear factor erythroid 2 like 2 (NFE2L2, encoding the transcription factor NRF2), resulting in constitutive NRF2 activation, are relatively common in SCC, with reported prevalences of 8%–35% depending on tumor site [15, 16] and are associated with aggressive tumor behavior, resistance to chemoradiotherapy and poor prognosis [17].

Phosphatidylinositol‐4,5‐bisphosphate 3‐kinase catalytic subunit alpha (PIK3CA) is reported as one of the most frequently altered genes in SCC with prevalence and clinical relevance varying by anatomical site but consistently representing a key molecular driver in SCC biology [15, 16, 18]. In a large multi‐organ SCC cohort, PIK3CA mutations were present in approximately 28.5% of cases, making it the second most common gene alteration after TP53. PIK3CA mutations are described as a defining feature of the “squamousness” molecular signature and are significantly more frequent in SCC compared to non‐SCC histologies [18].

In studies and reviews on the mutational landscape of SCC, Ankyrin 3 (ANK3) and FIG. 4 (Factor‐induced gene 4) are not reported as recurrently or clinically significant mutated genes [15, 16, 18].

Another notable finding was a previously undescribed SOX9 (SRY‐Box Transcription Factor 9) p.Thr243Pro missense variant, detected exclusively in the squamous component. The role of SOX9 and other genes of the SOX family as cell fate determiner during embryonic development and adult life by controlling progenitor cells to either proliferate or differentiate and therefore their potential role in neoplastic diseases and cancer cell plasticity, including cellular reprogramming, has been well characterized and the roles of SOX2 and SOX9 in SCC have been investigated in current literature [19, 20]. On the other hand, SOX9 mutations were not found as typical mutations in molecular studies of large cohorts of mesotheliomas [12, 13]. The detected variant p.Thr243Pro has not been reported in ClinVar or cBioPortal, suggesting it is extremely rare or novel. It lies within the transactivation middle (TAM) domain of SOX9. Because variants in the TAM domain have been shown to reduce SOX9 protein stability and transcriptional output, a destabilizing effect of p.Thr243Pro would be expected to decrease rather than increase SOX9 activity, which is not readily reconciled with a gain of squamous differentiation. We therefore regard p.Thr243Pro as a variant of uncertain significance whose functional consequence remains unknown, and report it as a novel, hypothesis‐generating observation. The squamous phenotype is more plausibly linked to the squamous‐associated drivers PIK3CA and NFE2L2 detected in the same component.

In conclusion, this case illustrates a rare variant of pleural mesothelioma with partial squamous differentiation diagnosed on limited biopsy material. Genomic profiling revealed a tumor mutational burden above the mesothelioma average and SCC‐associated mutations. Together, these findings support the concept that transdifferentiation in mesothelioma, though rare, may be driven by squamous‐associated genetic alterations in the context of increased genomic instability. These findings also highlight the diagnostic importance of combined histopathological, immunohistochemical and molecular assessment in unusual mesothelioma presentations and expand our understanding of tumor plasticity in this disease.

## Author Contributions

Julius Bodemeyer, Mara Elisa Mierzwa, and Hanibal Bohnenberger contributed in manuscript preparation and editing of the case. Jörg Seraphin, Rizkallah Maroun, Wolfgang Körber, and Christoph Engelke contributed in clinical diagnosis and management. Julius Bodemeyer, Hanibal Bohnenberger, Philipp Ströbel, Alexander Marx, and Felix Bremmer contributed in pathology diagnosis. Kirsten Reuter‐Jessen, Tessa Rosenthal, and Tim Heißenberg contributed in evaluation of the molecular results.

## Ethics Statement

The study was conducted under an ethics approval from the University Medical Center, Göttingen (ethic approval number 24‐4‐20) and have been performed in accordance with the ethical standards of the Declaration of Helsinki.

## Consent

Consent for publication was obtained from the patient and can be provided upon request.

## Conflicts of Interest

The authors declare no conflicts of interest.

## Supporting information


Supporting File 1

Supporting File 2

## Data Availability

The datasets supporting the conclusions of this article are included within the article and in the supplementary files. Additional file 1 (.pdf): WES mesothelial component (all detected variants in the mesothelial component). Additional file 2 (.pdf): WES squamous component (all detected variants in the squamous component).
