# Supplementary material for: Pleural Mesothelioma With Unusual Squamous Differentiation: A Clinicopathological Case Report With Molecular Insights From Whole‐Exome Sequencing
Source: Pathol Int. 2026 Oct 7;76(10):e70190. doi: 10.1111/pin.70190 (PMC13646670; doi:10.1111/pin.70190)
Supplement: Supplementary file 2 [file PIN-76-0-s002.pdf]

Molekularpathologische Untersuchung:
Next Generation Sequencing

Block / Präparat 1-1   Block / Präparat alt. / extern

Tumorzellgehalt 40   Labornummer DNA 25/R062   DIN   ng/µL

NGS Lauf	25/024	Qualitätsparameter	NGS:	Analyse-ID	26466
Sequenzierpanel	WES Tumor	Durchschn. Coverage	393		
Sequenziergerät	illumina NovaSeq	Total Reads	625748672		
Software	ICA Dragen v4	Median Insert size	60,5		

Tumormutationslast:

TMB Status TMB-low   TMB score 9,5

Mikrosatelliteninstabilität:

MSI Status nicht untersucht   MSI Loci   Instabile Marker   % instabil

Genomic Scars:

HRR Defizienz nicht untersucht   GIS Score   Grenzwert: 42   Ploidy

Varianten:

Gen	Exon	Variante	VAF	Transkript	HGVSc
VWA1	1/3	p.(Gly25ArgfsTer74)	0,41	NM_022834.5	c.62_71dup
FIG4	2/23	p.(Ile41Thr)	0,22	NM_014845.6	c.122T>C
BAP1	15/17		0,2	NM_004656.4	c.1891-6_1900del
ANK3	37/44	p.(Thr1861Met)	0,47	NM_020987.5	c.5582C>T
NFE2L2	2/5	p.(Gln26Pro)	0,32	NM_006164.5	c.77A>C

Molekularpathologische Untersuchung: Next Generation Sequencing

BAP1	4/17	p.(Ile76MetfsTer2)	0,29	NM_004656.4	c.228del
PROKR2	2/3	p.(Arg85His)	0,43	NM_144773.4	c.254G>A
PTPRD			0,54	NM_002839.4	c.5534+7_5534+8insACAGTTCAGGAA TGGTAAGTT
MKS1			0,49	NM_017777.4	c.1408-34_1408-6del
CHEK2	4/15	p.(Ile157Thr)	0,26	NM_007194.4	c.470T>C
PIK3CA	10/21	p.(Glu545Lys)	0,27	NM_006218.4	c.1633G>A
TBL1XR1	5/16	p.(Ile141SerfsTer3)	0,28	NM_024665.7	c.422_423del
ZFHX3	9/10	p.(Gln2564Arg)	0,43	NM_006885.4	c.7691A>G
HLA-B	4/8	p.(Arg243Trp)	0,06	NM_005514.8	c.727C>T
IL21R	8/9	p.(Arg275Gln)	0,45	NM_181078.3	c.824G>A
PRKN	5/12	p.(Arg191Trp)	0,4	NM_004562.3	c.571C>T
SPRTN	4/5	p.(Arg164Gln)	0,49	NM_032018.7	c.491G>A
TMEM41A	5/5	p.(Tyr196His)	0,48	NM_080652.4	c.586T>C
C5orf22	1/9	p.(Arg12Pro)	0,62	NM_018356.3	c.35G>C
MUC4	2/25	p.(Asp2357Asn)	0,48	NM_018406.7	c.7069G>A
MUC4	2/25	p.(Ala1897Glu)	0,16	NM_018406.7	c.5690C>A
MPL			0,14	NM_005373.3	c.1565+5C>T
DUSP22	7/7	p.(Pro195Leu)	0,33	NM_001286555.3	c.584C>T
GNAS	1/13	p.(Arg217His)	0,47	NM_016592.5	c.650G>A

MKKS	3/6	p.(Ala242Ser)	0,5	NM_170784.3	c.724G>T
PDE4DIP			0,18	NM_001198834.5	c.7000-7C>T
DRD4	1/4	p.(Ala79SerfsTer21)	0,39	NM_000797.4	c.235_247del
CEP89	17/19	p.(Ser643Gly)	0,45	NM_032816.5	c.1927A>G
PDE4DIP	1/47	p.(Thr6Ala)	0,10	NM_001395426.1	c.16A>G
BCR	19/23	p.(Val1094ArgfsTer17)	0,23	NM_004327.4	c.3275_3278dup
BCR	19/23	p.(Asp1106Asn)	0,1	NM_004327.4	c.3316G>A
ARID1A	20/20	p.(Asp1850ThrfsTer33)	0,58	NM_006015.6	c.5548del
KMT2C	8/59	p.(Asp348Asn)	0,06	NM_170606.3	c.1042G>A
GNAS	1/13	p.(Gln371Arg)	0,11	NM_001077490.3	c.1112A>G
PABPC1			0,05	NM_002568.4	c.1447+1_1448-1del
SMARCB1	6/9	p.(Asp251His)	0,37	NM_003073.5	c.751G>C
TNFRSF6B	1/3	p.(Arg103Gln)	0,5	NM_003823.4	c.308G>A
MUC16	3/84	p.(Met7950Arg)	0,45	NM_024690.2	c.23849T>G
GPX4			0,53	NM_002085.5	c.476+5G>A
SOX9	3/3	p.(Thr243Pro)	0,08	NM_000346.4	c.727A>C
CCT6B	14/14	p.(Ter531LeuextTer1)	0,5	NM_006584.4	c.1592G>T
SPECC1	11/15	p.(Arg937Leu)	0,38	NM_001243439.2	c.2810G>T
ZFHX3	9/10	p.(Met2102Thr)	0,5	NM_006885.4	c.6305T>C

PKD1	40/46	p.(Glu3786Gln)	0,48	NM_001009944.3	c.11356G>C
WDR90	30/41	p.(Gly1195Val)	0,49	NM_145294.5	c.3584G>T
PDS5B	31/35	p.(Ser1204Pro)	0,48	NM_015032.4	c.3610T>C
LTK	11/20	p.(Pro465Ter)	0,48	NM_002344.6	c.1393_1394del
TLL2	4/21	p.(Ala138Ser)	0,3	NM_012465.4	c.412G>T
PTK7	19/20	p.(Arg964His)	0,53	NM_002821.5	c.2891G>A
TUSC3	7/11	p.(Gln274Lys)	0,24	NM_006765.4	c.820C>A
TNFAIP3	7/9	p.(Gly456Trp)	0,66	NM_001270508.2	c.1366G>T
IQCB1	14/15	p.(Glu481Lys)	0,48	NM_001023570.4	c.1441G>A
CTNNB1	10/15	p.(Gly512Ala)	0,26	NM_001904.4	c.1535G>C
CAD	11/44	p.(Asp470Asn)	0,03	NM_004341.5	c.1408G>A
PRCC	3/7	p.(Pro185Leu)	0,36	NM_005973.5	c.554C>T
USP1	9/9	p.(Val737Met)	0,48	NM_003368.5	c.2209G>A
RBM10	9/24	p.(Pro275Ser)	0,99	NM_005676.5	c.823C>T
TAF1	1/38	p.(Asp7Gly)	0,53	NM_004606.5	c.20A>G
FURIN	16/16	p.(Arg754His)	0,56	NM_002569.4	c.2261G>A
AFDN	11/34	p.(Lys498Thr)	0,48	NM_001386888.1	c.1493A>C
NACA	3/9	p.(His1122_Pro1144del)	1	NM_001365896.1	c.3365_3433del

Molekularpathologische Untersuchung:
Next Generation Sequencing

Kopienzahlveränderungen:

Gen	Amplifikationsfaktor

nicht untersuch

Spezifikation illumina Twist Exom v2.5

Analysate	genomische DNA (z.B. aus FFPE-Gewebe, Zytopräparaten, Frischgewebe etc.)
Tumorzellgehalt	DNA aus FFPE-Gewebe: mind. 10%, für TMB/MSI >30%
Assay	illumina Twist Bioscience for Illumina Exome 2.5 Panel mit cfDNA Enrichment
Genomic Content	37,5 mega-Basen
OPS Code	Gewebe oder Zytopräparat: 1-996.08
Methode	NGS (Next Generation Sequencing): massive Parallelsequenzierung, Hybrid Capture Verfahren, Verwendung von Fall-spezifischen Indices, Verwendung von molekularen identifiern, intern zur Verwendung im diagnostischen Bereich validiert
Auswertung	illumina Dragen Pipeline: Read mapping, Varianten Calling (Referenzgenom: hg19), Zielwert durchschnittliche Coverage: >300x, Variantenbewertung illumina connected insights-unterstützt
Varianten	Es werden ausschließlich (wahrscheinlich) onkogene und pathogene Varianten berichtet.
